# Supplementary material for: Mod3D: A low-cost, flexible modular system of live-cell microscopy chambers and holders
Source: PLoS One. 2022 Jun 3;17(6):e0269345. doi: 10.1371/journal.pone.0269345 (PMC9165904; doi:10.1371/journal.pone.0269345)
Supplement: S1 File — (PDF) [file pone.0269345.s003.pdf]

Mod3D Live Cell Chambers and holders 3D printing and Assembly

and Ray Truant[1,2]
[1]Department of Biochemistry and Biomedical Sciences, McMaster University. Hamilton, Ontario, Canada, L8N3Z5;
[2]Center for Advanced Light Microscopy (CALM), McMaster University.

dx.doi.org/10.17504/protocols.io.n92ldz587v5b/v1

and Ray Truant

Live-cell microscopy imaging typically involves the use of high-quality glass-bottom chambers that allow cell culture, gaseous buffer exchange and optical properties suitable for microscopy applications. However, commercial sources of these chambers can add significant annual costs to cell biology laboratories. Consumer products in three-dimensional printing technology, for both Filament Deposition Modeling (FDM) and Masked Stereo Lithography (MSLA), have resulted in more biomedical research labs adopting the use of these devices for prototyping and manufacturing of lab plastic-based items, but rarely consumables. Here we describe a modular, live-cell chamber with multiple design options that can be mixed per experiment. Single reusable carriers and the use of biodegradable plastics, in a hybrid of FDM and MSLA manufacturing methods, reduce plastic waste. The system is easy to adapt to bespoke designs, with concept-to-prototype in a single day, offers significant cost savings to the users over commercial sources, and no loss in dimensional quality or reliability.

DOI

dx.doi.org/10.17504/protocols.io.n92ldz587v5b/v1

and Ray Truant . Mod3D Live Cell Chambers and holders 3D printing and Assembly. **protocols.io** https://protocols.io/view/mod3d-live-cell-chambers-and-holders-3d-printing-a-b7gtrjwn

NSERC Canada
Grant ID: RGPIN 6642-2020

protocol ,

Apr 12, 2022

Apr 12, 2022

60659

1 For FDM prints of holders, print at 20% grid infill with a layer height of 0.16 mm on either a Creality Ender 3 or a CR10 printer (Creality, Shenzhen, China) or similar FDM printer. Poly lactic acid (PLA) or Polyethylene terephthalate glycol (PETG) 1.75mm filament should be used. Acrylonitrile butadiene styrene (ABS) is not recommended as warping is an issue. Print nozzle is 0.4mm size or less and should be 195-205C, with heated bed at 60C, but should be optimized for the plastic being used.

2 For chamber holder tops and bottoms, include a >6mm brim on the bottom to ensure the print remains flat. A thin layer of polyvinyl alcohol (PVA) glue stick on the printer bed can be used to promote adhesion and prevent any warping.

3 To minimize warping, the print should not be removed from the heated print bed until cooled to room temperature.

4 Any PVA glue residue can be removed with a warm water wash.

5 Within the print, glue four neodymium 5x1mm magnets into place on each top and bottom half of the chamber holder with polyurethane or cyanoacrylate glue. Make sure to check that magnet polarity is correct to allow clamping and not repulsion. Allow glue to cure as per glue manufacturer instructions. Chamber printing (MSLA)

6 Use an Anycubic Photon printer (Anycubic, Shenzhen, China), or similar, with eSun PLA biocompatible resin LC1001 (Shenzhen eSUN Industrial Co.,China) using the standard settings for that printer located in the Chitubox software profile, with the exception of a 160 second first later exposure and a 30 second per subsequent layer exposure to ensure inverted print adhesion to platform. Glue surface should be the top or final layer of the print. Make sure the anti-aliasing option is NOT used in the mask profile.

7 Mix resin bottle well. Print in black resin with four 22x50mm chambers or eight 22x22mm chambers. Print chambers and lids directly on the printer platform without any rafts or support.

8 Printing should be done with light cover in place.

9 Printer and open resin should preferably be located within a chemical fume hood.

10 All handling of resin should be done with nitrile gloves, safety eyewear and a NIOSH organic carbon filter mask.

11 Clean up any spilled resin with 99% IPA and paper towels, or UV irradiate the area and chip off hardened resin for disposal. MSLA Post Processing

12 After printing, release 3D prints from the platform with a plastic scraper and wash for 15 minutes in fresh 99% isopropyl alcohol (IPA) in a Anycubic Wash & Cure Plus Machine (Anycubic, Shenzhen, China). Alternatively, prints can be washed in fresh IPA in a sealed polypropylene plastic container with vigorous shaking for 15 minutes. Thorough washing is essential to remove toxic free monomers and IPA should not be reused.

13 Prints are then cured under 405nm light in the same machine, for 30 minutes on the built-in rotating turntable. Alternatively, a 50W 385nm flood lamp (80mW/cm2) (WOWTOU, China) can be used: place face down on a 3D-printed box for 30 minutes, flipping once to avoid shadows. Any UV light source such as a UV light box can be used as an alternative, but move and flip the prints to avoid shadows.

14 Save used IPA for cleanup, but do not reuse the IPA for print washes. Chamber assembly to Coverslips

15 Use a 30mm diameter, 15cm wide craft roller to spread silicone RTV glue (SS-433T, Silicone Solutions, OH, USA) onto a 25X25 sheet of phenolic resin or glass plate.

16 Once the glue is spread evenly, use the roller to evenly transfer a thin layer of glue to the bottom surface of the inverted chamber.

17 With gloves on, gently press a 22x22mm #1.5 glass coverslip (VWR, USA) onto the glue face with a 3D printed tamper block (Supplemental Video 1). Visually inspect the glue surface to ensure complete contact and no voids.

18 Allow the glue to fully cure for at least 16hrs in the humid environment of a tissue culture incubator, to accelerate the curing time for RTV silicone. Alternatively, 72 hours without humidity incubation. Chamber Sterilization

19 In a HEPA tissue culture hood, bathe both chambers and lids 70% IPA for 10 minutes in a polypropylene tub, followed by filtered sterile water wash.

20 Remove from the bath and allow chambers and lids to dry under UV hood light and HEPA air flow on a sterile surface.

21 Place chamber and lid in a 5x10cm clear polypropylene bag under aseptic handling conditions with small sterile forceps.

22 UV irradiate the bagged chambers and lids prior to storage. Complete UV sterilization using either a 30mW/Cm2 365nm ELC-500 UV (ELC-500, Electro-Lite, USA) chamber on a rotating platform for at least 10 minutes per side, or the flood lamp curing chamber previously described for at least 30 minutes per side (see Fig. 3).

23 Repeat UV irradiation of the bagged chambers and lids prior to use. Do not attempt heat sterilization by autoclaving.
